# Hesperidin alleviates zinc oxide nanoparticle induced hepatotoxicity and oxidative stress

**DOI:** 10.1186/s40360-018-0256-8

**Published:** 2018-10-19

**Authors:** Sabah Ansar, Manal Abudawood, Amal S. A. Alaraj, Sherifa S. Hamed

**Affiliations:** 1 Clinical Laboratory Sciences, Applied Medical Science, King Saud University, Riyadh, Saudi Arabia; 2 Zoology Department, College of Science, King Saud University, Riyadh, Saudi Arabia; 3 Zoology Department, Faculty of Science, University of Alexandria, Moharram Bey, Alexandria, 21511 Egypt

**Keywords:** Antioxidant enzymes, Zinc oxide, Metals, Nanoparticles, Hepatotoxicity

## Abstract

**Background:**

Nanoparticles are widely utilized in many products such as cosmetics and sunscreens. The present study was undertaken to evaluate the effect of hesperidin (HSP) on nano zinc oxide particles (nZnO) induced oxidative stress in rat livers.

**Methods:**

Rats were randomly divided into 4 groups of 6 rats each and exposed to single administration of nZnO intraperitoneally (600 mg/kg bwt) and HSP (100 mg/kg bwt) by gavage. Group I served as the control; group II was given nZnO only; groups III received HSP only and group IV received nZnO 1 h after pretreatment with HSP for 7 days.

**Results:**

Compared to the controls, nZnO administration enhanced alanine aminotransferase (AST) and aspartate aminotransferase (ALT) levels (*p* < 0.05) with reduction in the levels of glutathione (GSH), catalase (CAT), glutathione peroxidase (GPx), superoxide dismutase (SOD) and increase in levels of malondialdehyde (MDA) while HSP attenuated nZnO-induced hepatotoxicity for above mentioned parameters.

**Conclusions:**

The induced toxicity in the liver was corrected by pretreatment with HSP. The findings of this study suggest that HSP pretreatment can potentially be used to prevent nZnO-induced biochemical alterations toxicity. Further, protection by HSP on biochemical results was confirmed by histopathological changes. The present study suggests that HSP can protect against nZnO-induced oxidative damage in the rat livers.

## Background

Nanoparticles are widely used in medical sciences and for the making of nano based drugs for some of the incurable diseases [1]. Even though zinc is an essential trace element and is commonly present in foods or added as a nutritional supplement, it has not got much attention during assessment of toxicity of nanoparticles. However, nano size particles can cause toxicity [2], because they are highly reactive and cause oxidative stress in human and animals as they can enter the circulation and reach to different organs of the body [3–5].

Earlier studies have reported that ZnO nanoparticles at a high dose of 1–5 g/kg can stimulate severe oxidative stress and cause apoptosis [6]. However, the toxicity of nZnO is more significant when the concentration is increased leading to toxic effects in different organs [7, 8]. nZnOs have been also shown to be toxic to microorganisms and rodents [9] and cause inflammation, altered heart rate and function, and oxidative stress in affected parts of the body [10, 11]. Once ingested, nanoparticles may be absorbed via intestinal lining and translocate into the blood stream and get metabolized in the liver [12]. Nanoparticles after uptake by the gastrointestinal tract get biodistributed in liver, kidney and spleen as the major organs [13].

Nanoparticles can cause oxidative stress and can lead to damage in protein structures and cause mutations [14]. Repetitive exposure to nZnO can induce DNA damage in human nasal mucosa and cause potential toxicity, including cytotoxic, genotoxic, and proinflammatory response [15, 16]. The increasing use of nonmaterial’s in healthcare and industrial products can lead to the possibility of their ingestion by humans, and other mammals. Titanium dioxide exposure can lead to toxicity and cellular responses of intestinal cells [17]. They may then translocate to blood causing adverse biological reactions in different tissues [18]. The adverse effects of silver nanoparticles (AgNPs) on the male reproductive tract, in particular spermatogenesis, and suggest that selenium can protect against AgNP-induced testicular toxicity [19].

Flavonoids are one of the most important antioxidants in fruits and vegetables, especially in the genus Citrus, as they provide health benefits through cell signaling pathways and antioxidant effects. Hesperidin (HSP, 3,5,7-trihydroxy-4-methoxy-flavanone-7-rhamnoglucoside) is a flavonone glycoside belonging to the flavonoid family acts as a potent antioxidant and anticancer agent [20]. HSP is largely isolated from citrus fruits and exhibits antioxidative, anti-inflammatory [21, 22], antihypercholesterolemic [23, 24], and antihyperglycemic [25] activities. In recent studies, beneficial effects of HSP against oxidative stress, cancer and obesity have been shown [20, 26–29]. Also, HSP significantly protected against hepatotoxicity induced by lipopolysaccharide [30], cadmium [31], acetaminophen [32], and carbon tetrachloride [33] toxicity in rats. Oxidative damage is implicated as a result of potential toxicity of nanoparticles; therefore, development of therapeutic agents with antioxidant properties is preferred. The molecular mechanisms of protection by HSP may include scavenging peroxynitrite radicals and inhibition of hydroxyl radical and reactive oxygen species (ROS). Our recent studies have shown that HSP enhances antioxidant defense with anti-inflammatory response against nZnO-induced neurotoxicity [34]. However, little is known on antioxidative properties of HSP associated with hepatotoxicity caused by nZnO. Henceforth, the objective of this study was to investigate the protective effect of HSP against nZnO-induced oxidative stress and hepatotoxicity.

## Methods

### Chemicals

HSP powder and ZnO nanoparticles less than < 100 nm particle size and composition of ZnO > 99.9% were purchased from Sigma-Aldrich, USA. ALT and AST kits and all other necessary reagents of analytical grade were bought from Sigma–Aldrich Chemicals Co., St. Louis, USA.

### Animals

Twenty-four male wistar rats weighing ~ 180–200 g were obtained from Pharmacy College, King Saud University. The experimental protocols were in compliance with the declarations of National Research Council [35]. Animals used in this study were placed in cages in an air conditioned room maintained with a 12-h light/ dark cycle. The rats were divided into four groups, each consisting of six animals as following groups; (1) Control rats (dimethyl sulfoxide was given as vehicle), (2) Rats received single administration of 600 mg/kg of nZnO, (3) Rats received oral administration of 100 mg/kg of HSP for 7 days, and (4) Rats received single administration intraperitoneally (i.p.) of 600 mg/kg of ZnO nanoparticles 1 h after pretreatment with HSP for 7 days. Oral administration took place by gavage. The dose of HSP and nZnO used in the present study were in accordance with previous reports, respectively [32, 36]. After 24 h of last treatment, the animals were euthanized (carbon dioxide was used as method of euthanasia) and blood samples were collected without using the anticoagulant and liver tissues were weighed and homogenized after adding saline. The supernatants were collected and assayed for different oxidative biomarkers.

### Histological examinations

Small pieces of liver tissue were fixed directly in 10% neutral buffered formalin.. Fixed tissues were dehydrated in ethanol, cleared in xylene, and embedded in paraffin. Tissues had been fixed in formalin for a month. Thin sections of 3 μm were cut, stained with haematoxylin–eosin (H&E) and examined under light microscope (Nikon Eclipse E600).

### Measurement of oxidative stress markers

For glutathione estimation free sulphydryl content was measured as described by Ellman [37] and expressed as mmole/mg of protein. For catalase activity (CAT) [38], hydrolysis of H2O2 was measured and absorbance was read at 240 nm. It was expressed as mM/mg protein. GPx activity [39] was monitored using H_2_O_2_ and the decrease of NADPH is proportional to GPx activity and expressed as nM/mg protein.

### Malondialdehyde (MDA) and superoxide dismutase (SOD) assay

Lipid peroxidation products were quantified by Draper and Hadley [40] method. In this reaction, the pink color obtained with MDA (an end product of lipid peroxidation) using TBA was determined at 535 nm wavelength. The results were expressed as micromoles per liter. SOD activity was determined according to the method of Sun Y et al. [41] described by measuring the auto-oxidation of pyrogallol at 440 nm. The results were expressed as units per liter.

### Statistical analysis

Results were analyzed using IBM SPSS software (22.0.0.0) and expressed as the mean ± standard error of the mean (SEM). One-way ANOVA of variance was applied to test for the significance of biochemical data of the different groups followed by Duncan’s multiple range test when appropriate. Results were considered significant when *p* ≤ 0.05.

## Results

### Intake and body weight measures

Data from these experiments showed no significant differences in food and fluid intake or body weight. Diet had no effect on mean body weight on any day.

### Effect on serum alanine and aspartate aminotransferase

**Fig. 1:**
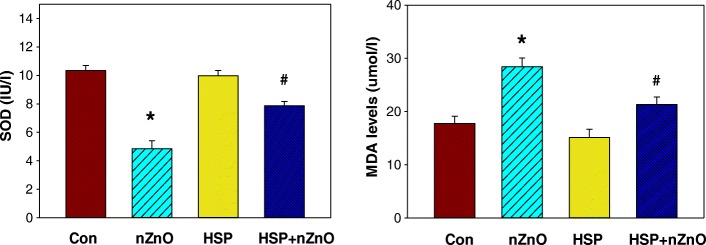
Effect of HSP on nZnO-induced on serum alanine aminotransferase (AST) and aspartate aminotransferase (ALT) levels. * *p* < 0.001 with respect to the control group. # *p* < 0.003 with respect to nZnO group and t value (AST: *t = 37.2; *P 0.001, DF = 1,11: #*t* = 70.5; #P 0.001, DF = 1,11: ALT: *t = 53.7; *P 0.001, DF = 1,11: #*t* = 43.74; #P 0.001, DF = 1,11)

Activities of AST and ALT were significantly increased in serum of rats treated with nZnO (*p* < 0.05) as shown in Fig. 1. However, administration of HSP in nZnO-treated group caused a significant reduction in their levels. The present study indicates that HSP supplementation normalized the AST and ALT activity in serum compared to nZnO treated rats. Pretreatment of the rats with HSP in nZnO-treated group caused a significant reduction in the levels of AST and ALT (*p* < 0.05). Activities of AST and ALT did not show any change in group 3 rats (HSP treated only) as compared to control group.

### Evaluation of biochemical parameters

Administration of HSP led to increase in the levels of GSH and antioxidant enzyme activities of GPx and CAT when compared with nZnO treated group, indicating HSPs enhanced capacity to scavenge lipid hydroperoxides. A significant reduction in levels of GSH was observed in nZnO treated group. However, a significant increase in GSH was seen in the group with pre HSP treatment. Also group exposed to nZnO showed a concentration-dependent statistically significant (*p* < 0.05) decrease in antioxidant enzyme activities of GPx and CAT. However, HSP pretreatment led to increase in enzyme activities (*P* < 0.05) as shown in Table 1.

**Table 1:** HSP protects ROS -mediated toxicity induced by nZnO

EXPERIMENTAL GROUPS				
Parameters	Control (I)	n ZnO (II)	HSP (III)	HSP + nZnO (IV)
GSH (mmol/mg prot)	1.08 ± 0.02	0.97. ±0.01^*^	1.12 ± 0.06	1.47 ± 0.04^**^
CAT (IU/mg prot)	1.95 ± 0.11	0.91 ± 0.14^*^	1.82 ± 0.11	1.45 ± 0.14^**^
GPx (U/mg prot)	1.13 ± 0.01	0.54 ± 0.07^*^	1.11 ± 0.05	0.95 ± 0.02^**^

All values are mean ± SEM, *n* = 6; * I vs. II (*P < 0.05). II vs. IV (**P < 0.05)

GSH: *t = 6.133; *P 0.002, DF = 5,11: **t = 4.45; **P 0.007, DF = 5,11/ CAT: *t = 49.2; *P 0.001, DF = 1,11: **t = 41.6; **P 0.001, DF = 1,11/ GPx: *t = 33.13; *P 0.002, DF = 1,11: **t = 43.74; **P 0.001, DF = 1,11

### Effect on superoxide dismutase (SOD) and malondialdehyde (MDA) levels

**Fig. 2:**
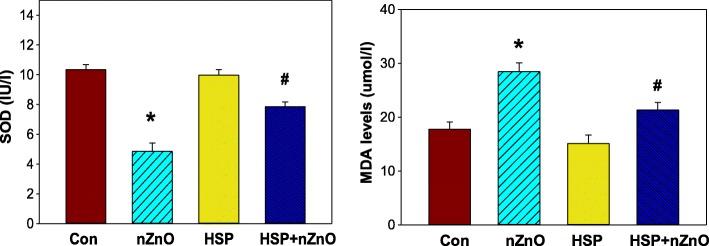
Effect of nZnO on the oxidative stress markers *(***a**) superoxide dismutase level (**b**) Malonaldehyde level. * *p* < 0.004, compared to control group. # *p* < 0.001, compared to nZnO group and t value (SOD: *t = 32.2; *P 0.001, DF = 1,11: # *t* = 78.1; # P 0.001, DF = 1,11: MDA: *t = 50.7; *P 0.001, DF = 1,11: #t = 70.5; #P 0.001, DF = 1,11)

One of the known toxic effects of free radicals is lipid peroxidation in membrane phospholipids related to the concentration of ROS generated. In this study, MDA and SOD levels were also measured. MDA levels were higher, and SOD levels were lower in nZnO treated group (*p* < 0.05 for MDA and *p* < 0.05 for SOD) when compared with those of the control group and recovery was significant in HSP pretreated group IV as compared to nZnO treated group II (*p* < 0.05 for MDA and *p* < 0.05 for SOD). However, there were no significant differences between the same parameters in HSP-alone treated group II and control group I (Fig. 2a, b).

### Histopathological effect

**Fig. 3:**
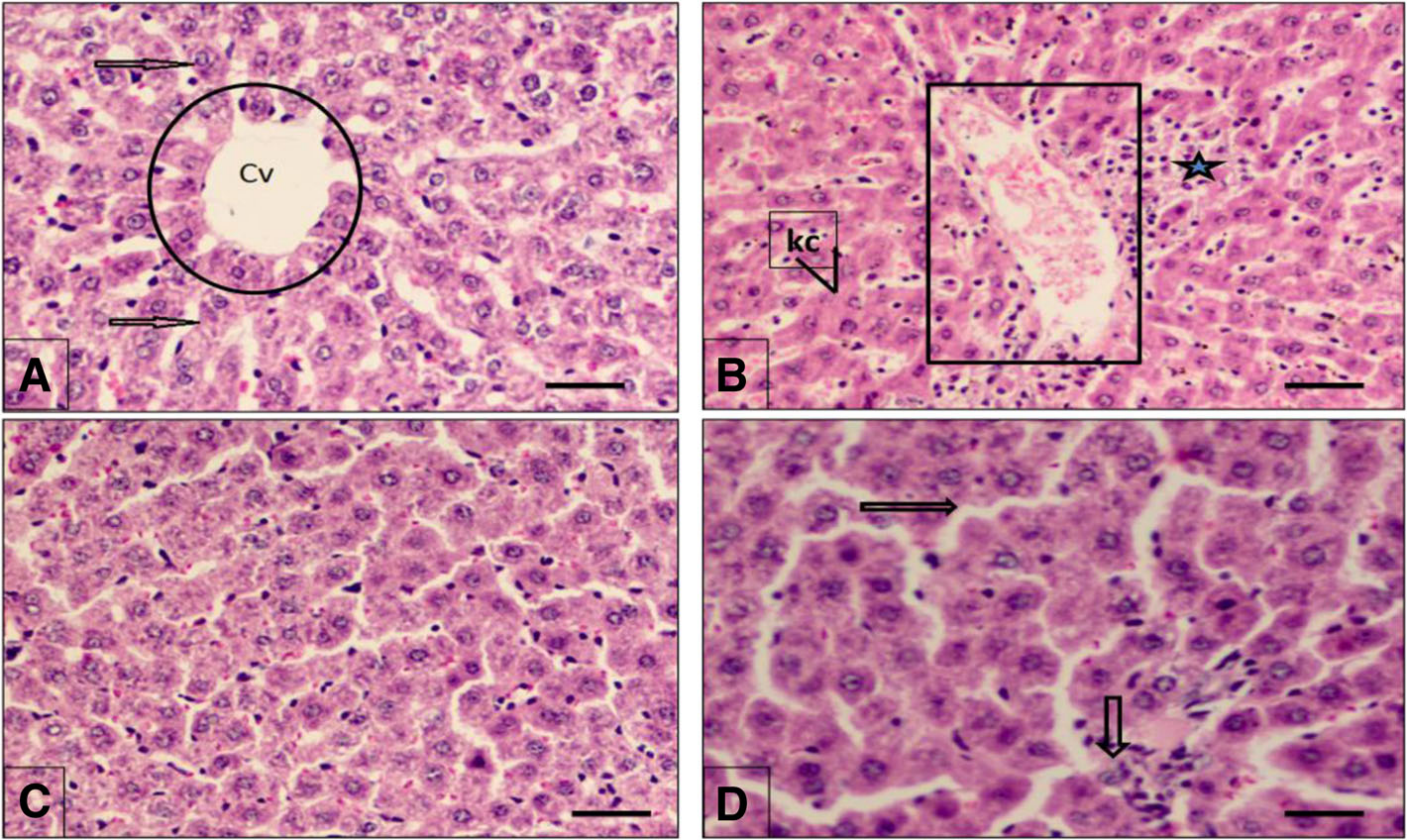
Effect of HSP and nZnO on the histology of rat livers: **a** Control group, showing normal liver architecture showing portal area (circle), hepatic strands of eosinophils (arrow), central vein (CV). **b** nZnO treated group showing portal tract expansion (square), hyalinization of hepatocytes, and dense infiltration of mononuclear cells (asterisks’), increase of Kupffur cell (kc), accumulation of cells around central vein. **c** Section of rat liver (HSP treated) showing normal hepatocytes and central vein (CV) similar to control. **d** Section of rat livers (HSP+ nZnO treated) showing normal cellular appearance and decrease in toxicity (arrow). Scale bar 50 μm. H & E stain

Microscopic examination of rat livers was performed and the histopathological analysis as shown in Fig. 3. (A) highlights the hepatic histology of the control rats showing no pathological changes and cells are normal in shape and size; (B) shows the abnormal changes in the liver due to nZnO induced stress including disorganization of normal radiating pattern of cell plates, and degeneration of normal histopathological hepatic cells. The histopathological profile of the rats treated with HSP showed no visible changes (C). The hepatic changes were protected in pretreatment with HSP in nZnO treated rats (D).

## Discussion

Nanoparticle exposure can damage liver which is a central organ in metabolism and detoxification and can be damaged by nanoparticle exposure [42]. Liver injury can change the level of liver enzymes such as AST, ALT which is an indicator of liver damage and liver diseases [43]. nZnOs are important metal oxide nanomaterials due to their various medicinal use and biological applications. Contact to nZnO induces oxidative stress and cytotoxicity in human colon carcinoma cells and induce neural stem cell apoptosis [44, 45]. Toxicity of nZnOs can be due to particle dissolution and may lead to reactive oxygen species (ROS) mediated damage [46, 47]. Also, intracellular ROS is mainly produced when the dissolved Zn2+ enters into the cells [48].

An important aspect of the nZnO use is the requirement that they should not be cytotoxic to the body. Previous studies showed that formation of ROS was responsible for cellular toxicity and the discharge of Zn + ions from the nZnOs, due to their instability in lysosomes which have acidic compartment leading to increase in the ROS generation [47].

Earlier studies have shown that nZnO exposure lead to MDA content increase in the zebra fish [49]. MDA is one of the main products of lipid peroxidation and can react with DNA bases [50]. It was observed earlier that oxidative effect of nZnO was much more than other non-metal nanoparticles [51]. Zhao et al. (2013) showed that nZnOs stimulates developmental toxicity, oxidative stress, and DNA damage on zebra fish embryos and may be partly due to the dissolved Zn^2+^ [49]. In this study, treatment with nZnO showed MDA levels higher, and SOD levels lower. However, pretreatment with HSP led to decrease in MDA levels and increase in SOD levels. Tissue lipid peroxide content is the most important marker of oxidative stress. HSP alone had no effect in this study given the basal levels of MDA are robust; indicating a detectable amount of lipid peroxidation is ongoing under basal conditions. HSP, which is an antioxidant and anti-inflammatory agent, could be that pretreatment with HSP protected the tissue against free radical damage. Reduction in the SOD level was also found on 24 h exposure of human embryonic kidney cells to nZnOs [52].

Exposure of endothelial cells to different nanoparticles causes tissue toxicity depending on its composition and concentration [53, 54]. Gojova et al. (2007) showed that nanoparticles stimulated inflammation and lead to production of ROS [53]. Also, it has been reported that exposure to 100 mg/L of nZnO caused a significant inhibition of SOD activity and more production of ROS and [49]. As levels of ROS produced by nZnOs exceed the capacity of cellular antioxidants, the antioxidant system cannot eliminate them. nZnO also has an inhibitory effect on CAT activity, which suggests that H_2_O_2_ generated by SOD cannot be removed completely by CAT directly and may cause intracellular buildup of ROS. Overall, we observed significant depletion in activities of antioxidant enzymes and GSH levels and significant increase in ALT, AST and MDA levels in rat liver tissue and this effect was reversed by the supplementation of HSP.

Recent histopathological studies demonstrated apoptosis in the pancreas, inflammation, edema, and retinal atrophy of the eye in the 500 mg/kg of nZnO treated group [55]. The present study showed abnormal changes including disorganization of normal radiating pattern of cell plates, and degeneration of normal hepatic cells in the liver due to nZnO induced stress. However, it was protected by pretreatment with HSP in nZnO treated rats. It has also been shown that nZnOs can cause various pathologic changes in the target organs with increased tubular deformations, necrosis and cytoplasmic vacuolations in the kidney, the edema, mononuclear cell infiltrations, and, the pyknotic nuclei in the liver [56].

## Conclusions

In conclusion, this study shows that HSP decreases liver toxicity in rats and that its effect is mediated by antioxidant activities. Results in this study suggest that HSP helps in reducing the hepatotoxicity of nZnO as also indicated by protection of histopathological changes in tissue. Future studies should examine the fact that HSP therapy targeted to liver toxicity may constitute an interesting strategy to reduce hepatotoxicity at molecular level.

## Abbreviations

AgNP: Silver nanoparticles
ALT: Alanine aminotransferase
AST: Aspartate aminotransferas
CAT: Catalase
DF: Degree of freedom
DMSO: Dimethyl sulfoxide
GPx: Glutathione peroxidase
GSH: Glutathione
HSP: Hesperidin
MDA: Malondialdehyde
nZnO: Nano zinc oxide particles
ROS: Reactive oxygen species
SOD: Superoxide dismutase
